# Cultivation of carbohydrate-rich microalgae with great settling properties using cooling tower wastewater

**DOI:** 10.1007/s11356-023-28432-w

**Published:** 2023-07-06

**Authors:** Edwin Ortíz-Sánchez, Rosa Angélica Guillén-Garcés, Sandra Morales-Arrieta, Patrick Ugochukwu Okoye, Hugo Olvera-Vargas, P. J. Sebastian, Dulce María Arias

**Affiliations:** 1 Universidad Politécnica del Estado de Morelos, Boulevard Cuauhnáhuac No. 566 Col. Lomas del Texcal, 62550 Jiutepec, Morelos, CP Mexico; 2 Instituto de Energías Renovables, Universidad Nacional Autónoma de México (IER-UNAM), Priv. Xochicalco s/n, Col. Centro, 62580 Temixco, Morelos, CP Mexico

**Keywords:** Algal biomass, Bioflocculation, Domestic wastewater, Cyanobacteria, Mineral accumulation

## Abstract

Wastewater treatment and simultaneous production of value-added products with microalgae represent a sustainable alternative. Industrial wastewater, characterized by high C/N molar ratios, can naturally improve the carbohydrate content in microalgae without the need for any external source of carbon while degrading the organic matter, macro-nutrients, and micro-nutrients. This study aimed to understand the treatment, reuse, and valorization mechanisms of real cooling tower wastewater (CWW) from a cement-processing industry mixed with domestic wastewater (DW) to produce microalgal biomass with potential for synthesis of biofuels or other value-added products. For this purpose, three photobioreactors with different hydraulic retention times (HRT) were inoculated simultaneously using the CWW-DW mixture. Macro- and micro-nutrient consumption and accumulation, organic matter removal, algae growth, and carbohydrate content were monitored for 55 days. High COD (> 80%) and macronutrient removals (> 80% of N and P) were achieved in all the photoreactors, with heavy metals below the limits established by local standards. The best results showed maximum algal growth of 1.02 g SSV L^−1^ and 54% carbohydrate accumulation with a C/N ratio of 31.24 mol mol^−1^. Additionally, the harvested biomass presented a high Ca and Si content, ranging from 11 to 26% and 2 to 4%, respectively. Remarkably, big flocs were produced during microalgae growth, which enhanced natural settling for easy biomass harvesting. Overall, this process represents a sustainable alternative for CWW treatment and valorization, as well as a green tool for generating carbohydrate-rich biomass with the potential to produce biofuels and fertilizers.

**Supplementary Information:**

The online version contains supplementary material available at 10.1007/s11356-023-28432-w.

## Introduction

The increasing adverse effects of global warming due to the current linear economic model needs the exploration and implementation of an alternative circular, clean, and sustainable economy. Such models involve waste recovery as an essential component. In this sense, municipal or industrial wastewater can be used as raw material to generate value-added products with the help of microorganisms such as microalgae and cyanobacteria. Such microorganisms have shown remarkable features, including high biomass growth rates, CO_2_ fixation capacity, and the production of biofuels and valuable by-products using wastewater as a substrate (Tiwari et al. 2019; Nilsson et al. 2020; You et al. 2022).

Recently, increasing consideration has been directed towards the development of sustainable biofuels from algal carbohydrates, such as bioethanol, biobutanol, and hydrogen gas, which represent an attractive alternative to reduce our dependence on fossil fuels. Some microalgae species can accumulate up to 65% of carbohydrates in terms of dry cell weight (dcw) in their cells, for which they are pondered as potential raw material for carbohydrate-derived biofuels (Xu et al. 2018; de Carvalho Silvello et al. 2022). Even the residual biomass from the carbohydrate extraction process (in the case of bioethanol production) could be considered as raw material for fertilizers under the concept of circular economy (Wuang et al. 2016; Ho et al. 2017).

However, there are still some drawbacks to be overcome, mainly (i) the low accumulation of carbohydrates in the biomass, which leads to low fermentation yields, and (ii) the energy-intensive harvesting process that represents high production costs (Nzayisenga et al. 2018; Arias et al. 2020; Wang et al. 2022). A high carbohydrate content in the biomass also carries additional benefits. For instance, Markou et al. (2012) related the bio-flocculation efficiency of *Arthrospira* sp. to an increase in carbohydrate accumulation. Greater settling efficiency could avoid the need for a costly harvesting process.

Microalgae store carbohydrates as glycogen, starch, amylopectin, sucrose, or even extracellular cellulose as a response of the chloroplasts to hostile environments, such as unbalanced carbon to nitrogen or phosphorus ratios, N and P limitations, or salinity changes (Arias et al. 2021). The utilization of wastewater as a source of nutrients to produce carbohydrate-rich biomass represents a promising approach to diminish production costs related to nutrient input (Gifuni et al. 2018; 2019). Recently, wastewater from different sources, such as domestic, urban, and agricultural, has been used to cultivate microalgae and produce carbohydrates (Arcila and Buitrón 2016; Shayan et al. 2016; Arias et al. 2020). Notably, the use of industrial wastewater containing high C/N and C/P ratios resulted in high carbohydrate content without any additional carbon supply (Sánchez-Contreras et al. 2021). However, because of the high organic and nutrient loads of industrial wastewater, it is generally necessary to dilute the influent or operate at high hydraulic retention times to unbalance the C nutrient ratios (de Farias Silva et al. 2020; Solís-Salinas et al. 2021). Recently, it was reported that the use of CO_2_ or bicarbonate also helped unbalance the C nutrient ratio and increased the carbohydrate levels up to 70% dcw (Rueda et al. 2020).

Following that last approach, cooling tower wastewater (CWW) from recirculating cooling or evaporative cooling water systems represents a potential carbon source to produce carbohydrate-rich biomass. CWW contains high alkalinity and suspended and dissolved solids (i.e., calcium phosphate, calcium carbonate) (Calderón et al. 2018; Soliman et al. 2022). It is used to remove heat from towers in several industrial processes (Garrido Arias et al. 2021). Once the cooling process is finished, a significant fraction of CWW is recirculated to the towers, while another portion is poured into the environment (Li et al. 2020b; Soliman et al. 2022). It must be noticed that besides the alkali compounds, CWW also contains ion oxides, magnesium silicate, and silica, among other pollutants. Therefore, this water needs a treatment to remove organic and inorganic chemicals to be applied either for recirculation to the towers or poured into the environment (Saha et al. 2020b). Different technologies, especially physical and chemical alternatives, have been tested for CWW treatment, for instance, reverse osmosis, electrodialysis, electrochemical oxidation, nanofiltration, and electrocoagulation (Li et al. 2020b; Saha et al. 2020a; Soliman et al. 2022). However, most of these processes are expensive and/or energy extensive, limiting their large-scale applications.

Low-cost options such as constructed wetlands were also applied for this purpose in the study of Saha et al. (2020b); however, this system required a supplementary treatment by electrochemical oxidation to eliminate macro- and micro-pollutants. Recently, photosynthetic-based approaches to treat CWW were proposed (Ortíz-Sánchez et al. 2022). It was demonstrated that CWW could naturally provide inorganic carbon and nutrient limitation to accumulate carbohydrates in batch cultivation; notwithstanding, the lack of macronutrients in CWW led to poor biomass growth and low productivity. Therefore, domestic wastewater must be included to promote biomass growth and carbohydrate content. Combining both effluents could be an exciting alternative since CWW can be mixed with sewage from the same industry to be treated by low-cost biological options. Although the results were promising, it was unclear if this treatment could be applied mid or long term to grow biomass and accumulate carbohydrates simultaneously. Besides, the removal of micronutrients (i.e., heavy metals) present in CWW was not analyzed, nor was its impact on microbial population and settling properties.

In this context, this study aims at understanding the mechanisms of treatment, reuse, and valorization of real CWW from a cement-processing industry mixed with domestic wastewater (DW) to produce carbohydrate-rich microalgal biomass with the potential for synthesis of biofuels or other value-added products. A cyanobacteria-dominated consortium was inoculated in three semi-continuous reactors to investigate the influence of HRT on C nutrient ratios, and their effect on microalgal growth, carbohydrate accumulation, and biomass settling properties. The impact of the wastewater on the content of micronutrients in the harvested biomass was discussed in detail.

## Materials and methods

### Stock culture

**Fig. 1:**
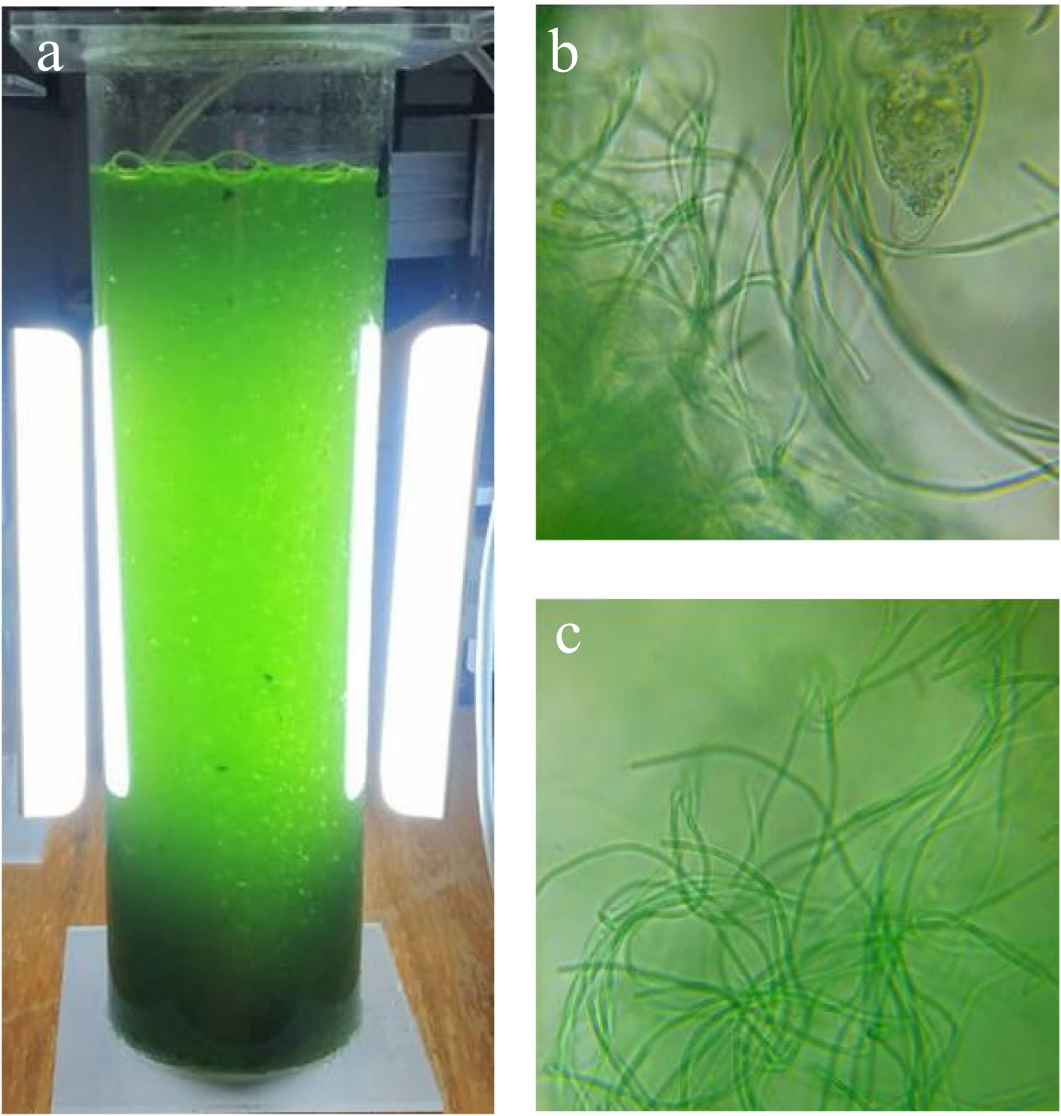
Inoculum used in this experiment: **a** suspended stock culture, **b** settled stock culture, and **c** microbial pictures taken at 1000× bright light microscopy

A mixed consortium of microalgae dominated by the cyanobacterium *Geitlerinema* sp. was isolated from a pond located in the city of Jiutepec, Morelos, Mexico (18.877420–99.165769) and identified in a previous study. The stock culture was kept in a cylindrical bubble-stirred photobioreactor (PBR) (10 cm diameter × 40 cm height) with an overall volume of 3.0 L and a working volume of 2.5 L with non-sterile BG-11 medium (Fig. 1). BG-11 was renewed weekly, allowing biomass settling for 30 min. The cultures were kept at temperatures between 13 °C and 31 °C, without pH control. Agitation was provided by an air pump (model A800, Hagen, Canada). The PBR was illuminated with two 12-W cold light LED lamps (model MLPF-40, Megaluz, Mexico) placed on both sides of each PBR at approximately 2 cm from the reactors that provided an average of 18,500 lx, experimentally measured within the reactor with a lux meter (Model HER-410, Steren, China). For inoculation, the stock cultures were settled in Imhoff cones for 30 min; then, the supernatant was removed. It should be noticed that the culture maintenance was performed under non-sterile conditions understanding that the object of this study is to use real wastewater which would imply changes in biomass population.

### Experimental setup

For the experiments, the same PBR shown in Fig. 1 was used. The photobioreactors were kept in light/dark periods of 12:12 h, without pH control, and were operated at room temperature (15–31 °C). The three PBRs were inoculated with 100 mL (2500 mg total suspended solids (TSS)) of thickened microalgae diluted with 2.4 L of tap water. As the experiment progressed, the PBRs were fed with a mixture of 75:25 v/v of CWW and DW. CWW was obtained from a nearby cement company, and domestic wastewater (from WC and kitchen) was obtained from a local wastewater treatment plant. The characteristics of the effluents are presented in Table 1. The dilution rate used in this study was chosen based on preliminary batch studies. (Supplementary material A1).

**Table 1:** Values of the main parameters of the domestic wastewater (DW) and the cooling wastewater (CWW) used in this experiment

Parameter	DW	CWW	DW + CWW
pH	7.23 (0.38)	8.6 (0.32)	8.36
Alkalinity (mg CaCO_3_ L^−1^)	386 (-)	470 (-)	464
Chemical oxygen demand (COD) (mg O_2_ L^−1^)	853.77 (109.19)	87.5 (4.95)	252.6
Total ammoniacal nitrogen (TAN) (mg L^−1^)	79.26 (1.18)	0.03 (0.01)	20.1
Nitrates (mg N-NO_3_^−^ L^−1^)	35.5 (-)	12.4 (-)	16.3
Nitrites (mg N-NO_2_^−^ L^−1^)	0.13 (-)	3.29 (-)	2.5
Phosphates (mg P-PO_4_^3−^ L^−1^)	22.8 (20.82)	4.85 (2.49)	8.02
Al (mg L^−1^)	0.14 (0)	0.10 (0)	0.11
Cd (mg L^−1^)	0.10 (0)	0.01 (0)	0.03
Cr (mg L^−1^)	0.03 (0)	0.00 (0)	0.01
Cu (mg L^−1^)	0.00 (0)	0.03 (0)	0.02
Fe (mg L^−1^)	4.48 (3.77)	1.78 (8.77)	2.45
Mn (mg L^−1^)	0.00 (0)	0.01 (0)	0.01
Ni (mg L^−1^)	0.11 (0)	0.04 (0)	0.06
Pb (mg L^−1^)	0.95 (0.02)	0.51 (0)	0.62

Each reactor worked with a different HRT: 10, 8, and 6 days. The corresponding volumes were calculated using Eq. 1.

1 $$TRH=\frac{Q}{V}$$

where *Q* is the flow (L day^−1^) and *V* is the total volume of the photobioreactor (L).

The PBRs were fed every day at the end of the dark phase considering a volume of 2.5 L; the amount of mixing liquor extracted from each reactor depending on the HRT was 250, 312.5, and 416.7 mL, for HRT of 10, 8, and 6 days, respectively. The lost volume was replaced with the 75:25 v/v CWW/DW mixture. The three PBRs were operated simultaneously for 55 days to ensure complete tap water removal and to reach metabolic stability.

### Analytical methods

#### Nutrients, carbon, and metal analysis

The supernatant from the PBRs (corresponding to the mixed liquor) was analyzed after settling. Total phosphorus was measured by colorimetry using a UV-Vis spectrophotometer (model UV-1900i, SHIMADZU, Japan) (APHA-AWWA-WPCF 2017). Total ammoniacal nitrogen (TAN) was determined by colorimetry with a phenol-sulphuric acid method (Solórzano 1969) using the same spectrophotometer. Alkalinity was measured by acid-base titration with H2SO4, and standardized sodium carbonate solution, while COD was determined by the closed reflux colorimetric method using a HACH DR 900 colorimeter. Both alkalinity and COD methods were measured according to standard methods (APHA-AWWA-WPCF 2017). N-NO3^−^, N-NN-NO_3_^−^, and N-NO_2_^−^ were determined with the Nitraver and Nitriver kits (HACH® Company) using the same HACH colorimeter. All these analyses were performed once per week, except by pH, which was measured twice per week with a WD-35610-10 potentiometer (OAKTON, USA).

The nutrient removal rate (*Tr*_*N*_) was calculated from Eq. 2.

2 $${Tr}_N=\frac{X_f-{X}_i}{\Delta T}$$

where *X*_*i*_ and *X*_*f*_ are the initial (mg L^−1^) and final concentrations of the nutrient analyzed, respectively, and ∆𝑇 is the time difference between the measurements (days).

Al, Cd, Cr, Cu, Fe, Mn, Ni, Pb, and Zn were measured by inductively coupled plasma atomic emission spectroscopy (Horiba Scientific ICP-AES, Japan).

#### Biomass growth and composition analysis

TSS and volatile suspended solids (VSSs) were measured gravimetrically in the mixed liquor (APHA-AWWA-WPCF 2017).

Samples for determination of chlorophyll *a* were collected once per week and centrifuged at 3000 rpm for 10 min. Then, it was collected in Eppendorf tubes and stored in a − 20 °C freezer. They were further processed in a lyophilizer at − 47 °C (Labconco Freezone 6, USA) for 48 h. Chlorophyll *a* was quantified according to (Ritchie 2008). Briefly, 5 mg of dry biomass with 10 mL of 90% methanol was added to 10 mL tubes. The tubes were heated at 75 °C in a water bath for 20 min; they were then centrifuged at 4500 rpm for 15 min at 4 °C. A 5 mL sample was taken from the supernatant and measured at 663 nm and 645 nm in a UV-Vis spectrophotometer (model UV-1900i, SHIMADZU, Japan). Chlorophyll *a* (mg g^−1^) was calculated following Eq. 4.

3 $$\textrm{Chl}\ a\ \left(\textrm{mg}\ \textrm{g}-1\right)=\frac{\left[\left(12.7\times \textrm{A}663\right)\hbox{--} \left(2.6\times \textrm{A}645\right)\right]\times \left({V}_m\ \right)}{W_b}$$

where 12.7 and 2.6 are constants and *A*_663_ and *A*_645_ are the absorbance readings at the wavelengths of 663 and 645 nm, respectively. *V*_*m*_ is the volume of methanol used for the analysis (mL), and *W*_*b*_ is the weight of the biomass used in the analysis (mg).

Microalgal samples were observed under an optical microscope (T360B, Amscope, USA) to assess microalgae and other microorganisms’ populations qualitatively. Since filamentous cyanobacteria cannot be properly quantified by the Neubauer chamber or related methods, quantitative analysis was not performed. However, the dominance of species was determined by observations of the culture carried out once per week taking samples of 25 μL in triplicate and placed on a slide and observed at 100×, 200×, and 1000×.

Relevant cell microalgae and grazers observed in more than one observation were isolated, and their genomic DNA was extracted for molecular identification according to the method of Laird et al. (1991). The internal transcribed spacer (ITS) region and 18s rDNA sequence were amplified by PCR techniques in an Applied Biosystems 2720 thermal cycler using Green Taq DNA polymerase (Thermo Scientific) and Phusion High-Fidelity DNA Polymerase (Thermo Scientific) and primers ITS1, ITS-4, NS3, and NS8. Later, the PCR mixture was purified and subsequently sequenced as explained elsewhere (Sánchez-Contreras et al. 2021). In all cases, biological reagents were used following the instructions provided by the supplier. Representative ITS region and 18s rDNA sequences were compared to the GenBank database of sequences present at the National Center for Biotechnology Information using the BLASTN algorithm (http://www.ncbi.nlm.nih.gov/BLAST).

#### Carbohydrate analysis

Carbohydrate accumulation was quantified by the phenol-sulfuric acid colorimetric method (DuBois et al. 1956) using a UV-Vis spectrophotometer (model UV-1900i, SHIMADZU, Japan). The biomass was centrifuged at 3000 rpm for 10 min, collected in Eppendorf tubes, and stored in a − 20 °C freezer, then lyophilized at − 47 °C (Labconco Freezone 6, USA) for 48 h. Then, 2 mg of dried biomass was hydrolyzed with 2 mL of 1 N HCl and left to react for 2 h at 100 °C. Carbohydrate production (*P Carbs*) was calculated using Eq. 4.

4 $$P\ Carbs={Pb}_t\times C\ {carbs}_t$$

where *Pb*_*t*_ is the biomass production at time *t* (g L^−1^) and *C carbs*_*t*_ is the carbohydrate content at time *t*.

#### Floc length analysis

Floc length of the culture was determined at the beginning and when the steady state was reached. A homogeneous sample of 20 mL was carefully taken from the mixed liquor of each reactor. The largest floc in the sample was taken with dissecting forceps and placed in a 5-cm-diameter petri dish, which was placed on a millimeter paper. Ten repetitions for each HRT were performed, and the results were averaged.

#### EDX analysis

Elemental analysis of the dried thickened biomass was obtained by scanning electron microscopy (SEM) measurements performed on an S-5500 microscope (Hitachi), and energy-dispersive X-ray spectroscopy (EDX) with a Brucker 133 V.

#### Statistical analysis

Data of the biomass concentration (VSS), flocs size, chl *a*, and carbohydrate production was analyzed to determine normality using a Shapiro-Wilk test and were normalized when necessary. Normalized data were then submitted to a one-way analysis of variance (ANOVA) with repeated measurements. *F* test was conducted at a 95% confidence to evaluate the model adequacy. Also, the correlation model variables was analyzed through the Pearson correlation coefficient.

## Results and discussion

### General performance

**Fig. 2:**
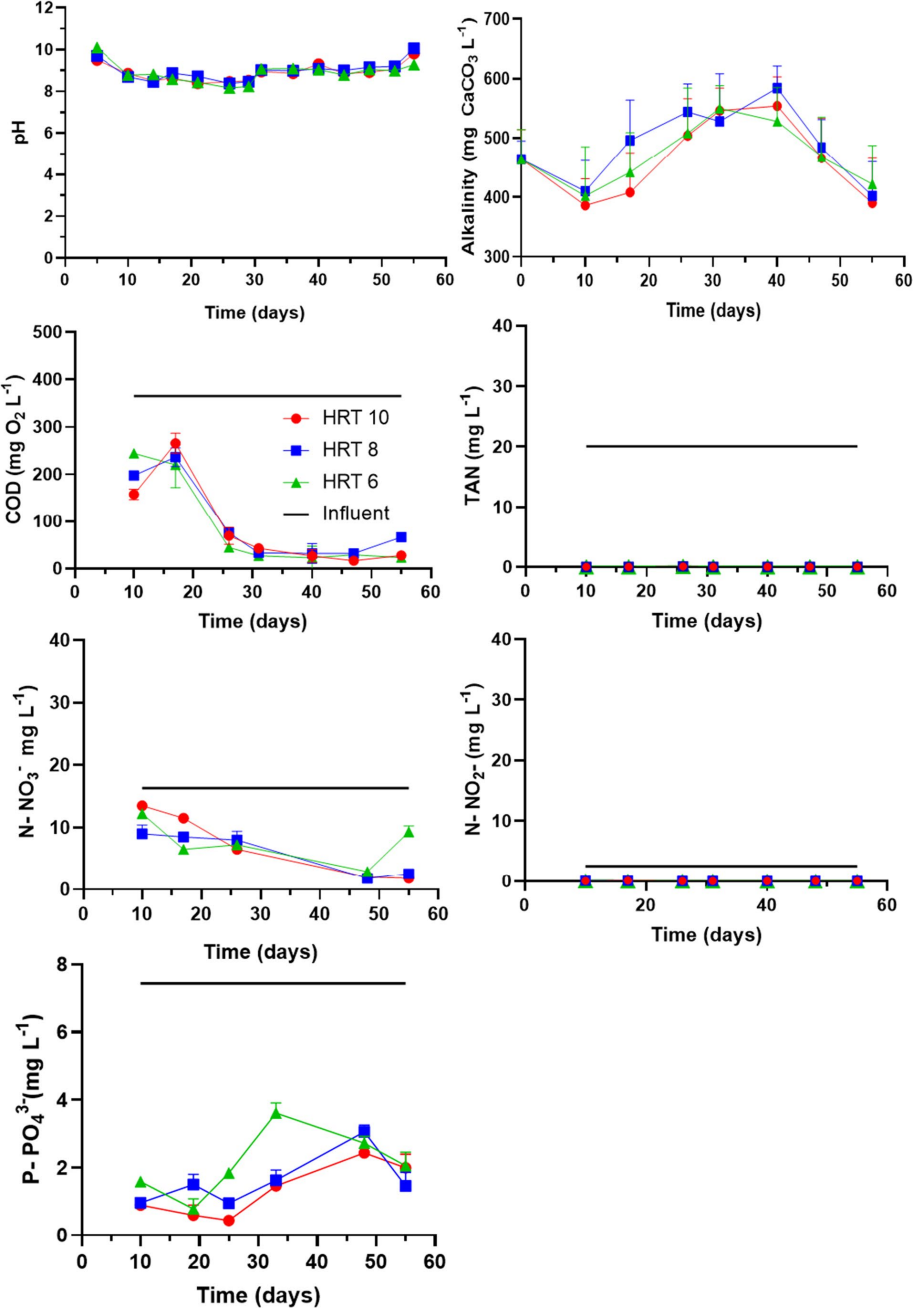
pH, alkalinity, chemical oxygen demand (DQO), and nutrients (total ammoniacal nitrogen (TAN), NO_3_^−^, NO_2_ and P-PO_4_^3−^ profiles of the photobioreactors working at 10, 8, and 6 days of HRT fed with 25% of domestic wastewater and 75% of cooling wastewater (DW25%-CWW75%))

The results obtained during microalgal cultivation using the DW25%-CWW75% mixed wastewater are presented in Fig. 2 and Table 2. In all cases, the pH values ranged from 8 to 10 in all the reactors. The average pH value in each reactor was 8.87, 8.95, and 8.86 for the operations at 10, 8, and 6 days of HRT, respectively. It should be noted that the pH of microalgae cultures for production purposes is in a range between 7 and 9 with an optimal operating value of 8.2 to 8.7 (Beltrán-Rocha et al. 2017).

**Table 2:** Average (standard deviation) of the main nutrient’s concentrations of the effluent (mixed liquor) of the photobioreactors operated at 10, 8, and 6 days of HRT

Parameters	Units	HRT 10	HRT 8	HRT 6
Alkalinity	Concentration (mg CaCO_3_ L^−1^)	464.86 (72.02)	492.57 (67.53)	474.29 (55.97)
	Removal rates (mg L day^−1^)	1.34 (-)	1.13 (-)	0.76 (-)
	Removal efficiency (%)	15.9(-)	13.36(-)	9.05(-)
Chemical oxygen demand (COD)	Concentration (mg O_2_ L^−1^)	86.48 (92.47)	96.11 (85.15)	82.26 (83.07)
	Removal rates (mg L day^−1^)	6.14 − 7.52	5.44 − 8.62	6.22 − 3.77
	Removal efficiency (%)	92.43 − 2.06	81.85 − 2.36	93.7 − 1.03
Total ammoniacal nitrogen (TAN)	Concentration (mg L^−1^)	0.03 (0.03)	0.03 (0.03)	0.04 (0.03)
	Removal rates (mg L day^−1^)	0.37–0.01	0.365 − 0.01	0.365 − 0.01
	Removal efficiency (%)	> 99	> 99	> 99
P-PO_4_^3−^	Concentration (mg L^−1^)	1.31 (0.80)	1.60 (0.77)	2.11 (0.97)
	Removal rates (mg L day^−1^)	0.10 (-)	0.11 (-)	0.10 (-)
	Removal efficiency (%)	73.1 (-)	80.25 (-)	72.26
N-NO_3_^−^	Concentration (mg L^−1^)	5.77 (5.48)	4.62 (3.45)	6.07 (3.38)
	Removal rates (mg L day^−1^)	0.27 (-)	0.26 (-)	0.24 (-)
	Removal efficiency (%)	90.18	87.12	47.24

Alkalinity was used as an indirect measure of inorganic carbon available in the medium. Remarkably, the CWW75%-DW25% provided enough alkalinity, maintaining values up to 350 mg CaCO_3_ L^−1^ during the experiments, without statistical differences among the photobioreactors (*P* = 0.08). It must be highlighted that HCO_3_^−^ predominates in the pH interval from 6.36 to 10.33, which makes alkalinity available for microalgae and carbohydrate accumulation. On the other hand, organic carbon measured as COD showed different patterns, and statistical difference among all the experimental conditions (*P* < 0.0001). During the first 20 days of operation (adaptation phase), the effluents of all the reactors registered concentrations up to 270 mg O_2_ L^−1^. Subsequently, COD concentration dropped to 50 mg O_2_ L^−1^ in 30 days, while remaining unchanged for the rest of the experiments. This represented a COD removal of > 80%. Interestingly, this culture did not show a COD increase as previously reported with industrial wastewater (Van Den Hende et al. 2014; Sánchez-Contreras et al. 2021). These previous results indicated that the culture possesses the capacity to assimilate organic compounds during the night without the addition of activated sludge to enhance COD removal as commonly practiced (Lee et al. 2019). Furthermore, the wastewater used in this study could be adding additional autotrophic and heterotrophic bacteria which could work symbiotically with microalgae to clean wastewater. This phenomenon inevitable with real unsterile processes could improve the efficiency of gas exchange (CO_2_ and O_2_), and the velocity of the bacteria to remove chemical oxygen demand (COD) with respect to algae (Chan et al. 2022).

In the case of the nutrients, P-PO_4_^3−^ showed a similar trend in all the reactors in the first 25 days, averaging 1.1 mg L^−1^. Subsequently, the concentration increased in the three reactors reaching values up to 3 mg L^−1^ in all the conditions at the end of the experiments, which represents more than 75% of removal efficiencies. Statistical analysis showed a significant difference between the photobioreactors (*P* < 0001). For nitrogen, TAN and N-NO_2_^−^ were completely consumed in all cases, remaining below the detection limit during the experiments. The concentration of N-NO_3_^−^ showed a different pattern; it was almost completely consumed (> 87% removal) at 10 and 8 days of HRT, whereas only 50% was removed at 6 days of HRT. Also, ANOVA showed significant differences (*P* < 0.0001). The complete uptake of TAN was expected since N-NH_4_^+^ is the preferred nitrogen form consumed by most microalgal and cyanobacterial species, followed by N-NO_3_^−^ and finally N-NO_2_^−^. These trends have been reported in several studies that used diluted and undiluted digestate (Arashiro et al. 2020; Fathima et al. 2020). Although microalgae could be assumed to uptake most of NH_4_^+^, NO_3_^−^, NO_2_^−^, and PO_4_^3−^, however, the presence of nitrifying/denitrifying bacteria in the microalgae-bacterial consortium may have also contributed to the N and P consumption. Other mechanisms as NH_3_ volatilization and P precipitation could also contribute to this phenomenon at high pH (Hwang et al. 2016; Ahmed et al. 2021).

The removal efficiencies obtained in this work are in good agreement with previous studies with semi-continuous cultures utilizing industrial effluents for microalgal cultivation. For instance, Sánchez-Contreras et al. (2021) removed more than 90% of P-PO_4_^3−^, and 50–80% of TAN, while the COD only decreased by 2% in 10 days of HRT. This study used mixed industrial wastewater from an industrial park to grow a culture dominated by cyanobacteria. In another investigation with wastewater from the aquaculture industry to feed *Tetraselmis suecica*, Andreotti et al. (2020) removed 97.18% of P-PO_4_^3−^ and 99.82% of TAN at 10 days of HRT in a semi-continuous reactor, while and 92.25% of P-PO_4_^3−^ was removed in 7 days.

Regarding the removal of heavy metals, Al, Fe, and Pb presented the highest concentrations in the influent (Table 1). All the photoreactors showed complete elimination of Al, while Fe was removed by 57.9, 56.3, and 56.7% at 10, 8, and 6 days of HRT, respectively. The removal of Pb was 30.6, 33.8, and 45.1%, respectively, at 10, 8, and days of HRT (Supplementary material A2). In all cases, the concentrations of the analyzed metals at the end of treatment were below the permissible limits established by the Mexican regulations (NOM-001-SEMARNAT 1996) (Supplementary material A2). The temperature has less impact on the removal of metals. However, the pH is an important factor in metal biosorption, because it influences the redox potential, dissociation state of binding sites, and ionic conditions of metals (Ahmed et al. 2021; Nateras-Ramírez et al. 2022). The elimination of these compounds from wastewater represents great difficulties since heavy metals and metalloids are generally non-biodegradable with a tendency to bioaccumulate, which causes serious human health and environmental issues (Pavithra et al. 2020; Ahmed et al. 2021).

The mechanisms through which microalgae remove heavy metals involve biosorption and bioaccumulation. Because the cell wall of microalgae is composed of polysaccharides, lipids, and proteins, many functional groups bind to heavy metals favoring adsorption. Bioaccumulation is a slower process during which metal ions are transported through the cell membrane into the cytoplasm, where they diffuse to bind to proteins and peptides, such as glutathione, metal transporters, oxidative stress reducing agents, and phytochelatins (Leong and Chang 2020).

Overall, the results of this experiment are similar to that reported by Torres et al. (2017), where they obtained removal efficiencies of 66% for total Fe in a treatment system with microalgae from the Chlorophyta division to remove the pollutant load of sewage from a coal mine. Also, Abdel-Razek et al. (2019) evaluated the use of pure cultures of *Chlorella vulgaris*, *Scenedesmus quadricuda*, and *Spirulina platensis* to eliminate Cd, Ni, and Pb from urban and agricultural wastewater. The microalgae used in this study were able to bioaccumulate nickel with an efficiency of up to 95%. Furthermore, those microalgal strains showed a remarkable absorption of Pb and Cd with an efficiency of 89% and 88%, respectively.

### Biomass growth and composition

**Fig. 3:**
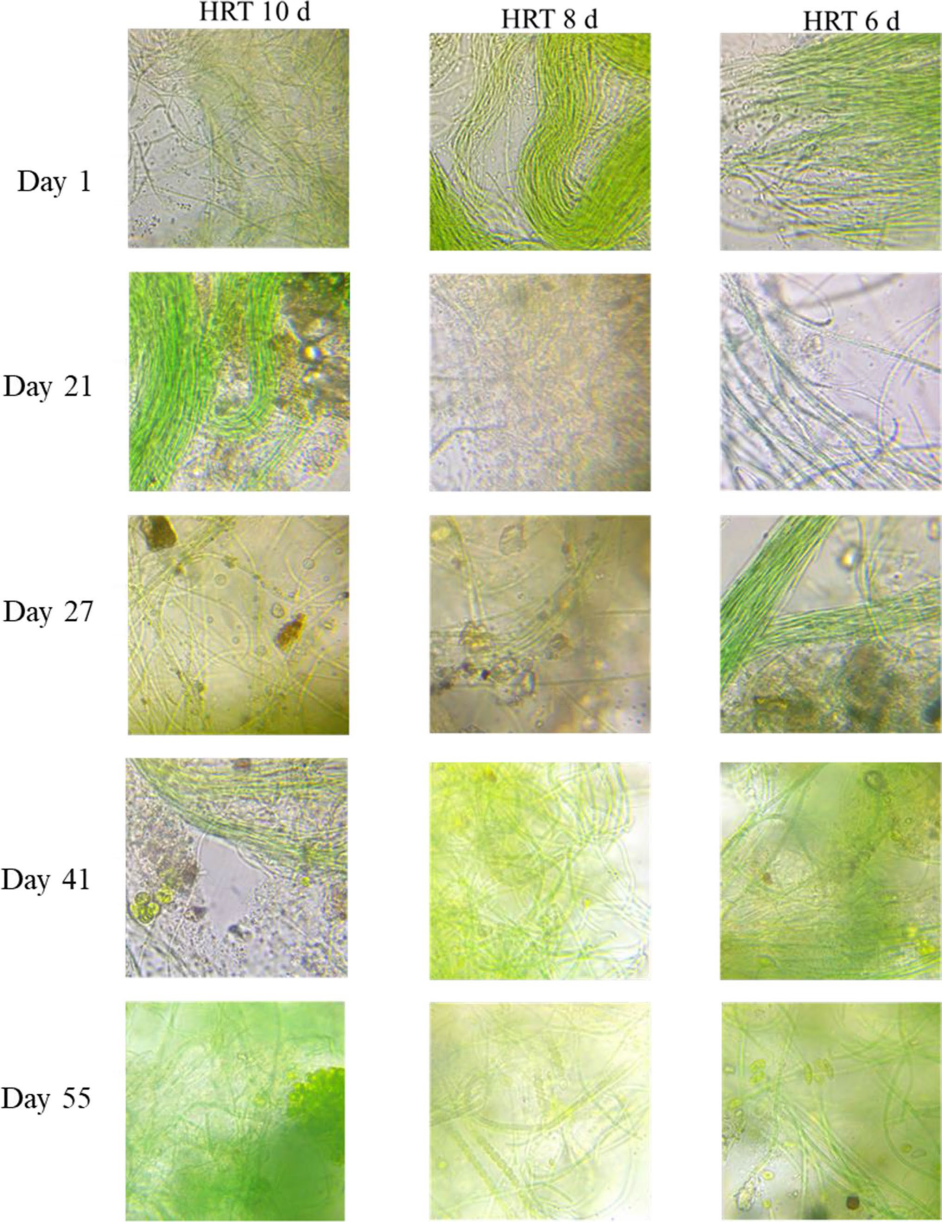
Micrographs of the inoculum observed in bright light microscopy at 1000× in photobioreactors operated at 10, 8, and 6 days of HRT fed with 25% of domestic wastewater and 75% of cooling wastewater (DW25%-CWW75%). Algal flocs and dispersed cells are mainly composed of cyanobacteria *Gleiterinema* sp., *Nostoc* sp., and green algae *Coelastrum proboscideum*, *Chlorosarcinopsis eremi*, and *Scenedesmus* sp.

Since all the experiments were operated under unsterile conditions, algal population experimented composition changes over time, although it remained dominated by filamentous cyanobacteria (Fig. 3). Other cyanobacteria species such as N-fixing *Nostoc* sp. were only barely observed. The identification at the molecular level of selected microorganisms species involved sequencing of specific probes in the genome, which must be unique to determine the genus. Within these probes or markers, 18 rDNA and the internal transcribed spacer 2 (ITS) have been used for the identification of microalgae (Radha et al. 2013). ITS of the nuclear ribosomal operon has been widely used as a barcode in algae and land plants (Moniz and Kaczmarska 2009) and green algae (Chirakkara et al. 2016), thus allowing to establish phylogenetic relationships and preliminary characterization.

**Fig. 4:**
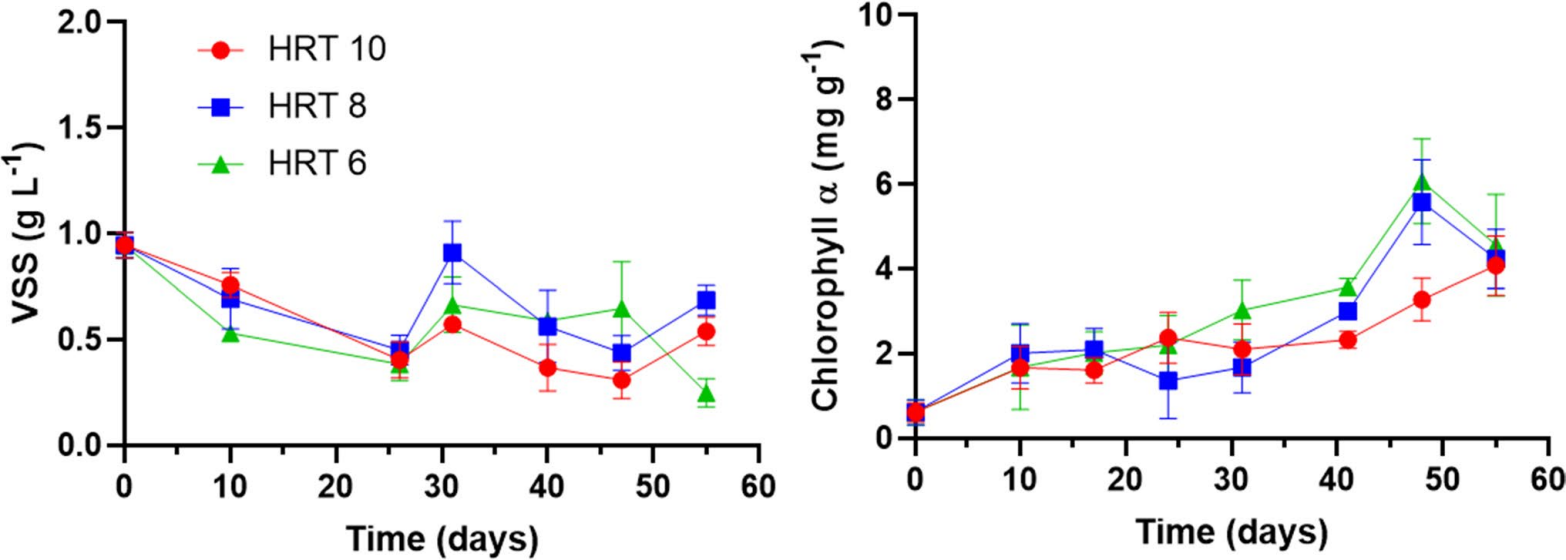
Biomass profiles measured in terms of volatile suspended solids (VSSs) and chlorophyll *a* in photobioreactors operated at 10, 8, and 6 days of HRT fed with 25% of domestic wastewater and 75% of cooling wastewater (DW25%-CWW75%)

The ITS region (800 bp) of the isolate was analyzed using the GenBank database, and the sequence similarity was analyzed using BLASTN (http://blast.ncbi.nlm.nih.gov/Blast.cgi). According to the BLASTN search using the ITS sequence as a query, the preliminary related species was *Coelastrum proboscideum* (97.51% identity), and *Chlorosarcinopsis eremi* (98.29% identity) appeared inside the flocs with dispersed *Scenedesmus* sp. (97.51% identity). Rotifers identified as *Ascomorpha ovalis* (86.17% identity). Biomass measured as VSS in the photobioreactors decreased during the first 27 days, from initial 0.9 g L^−1^ to 0.4, 0.45, and 0.38 g L^−1^ at 10, 8, and 6 days of HRT, respectively. The operation at 10 and 8 days maintained a steady state averaging 0.4 ± 0.1 and 0.6 ± 0.1 g L^−1^ from day 27 to day 55 (Fig. 4). In contrast, the photoreactor at 6 days of HRT maintained a constant biomass concentration of 0.6 ± 0.2 g L^−1^ from day 27 to day 47 and then dropped to 0.2 g L^−1^ on day 55. The highest biomass concentration was reached at 8 days of HRT on day 31, reaching 0.91 g L^−1^, with a production of 0.1 g L^−1^ day^−1^. Remarkably, any photoreactor presented a significant difference among them (*P* > .05) in biomass production.

Regarding chlorophyll *a* content, all the conditions showed an increasing pattern during the first 10 days of cultivation; afterward, they maintained steady until day 30. Subsequently, they increased in the last days of operation, reaching the highest pigment content of 5.3 and 5.7 mg g^−1^ at HRT of 8 and 6 days, respectively (Fig. 4), without significant differences among them. The color disparity between micrographs presented in Fig. 3 contrasting with chlorophyll *a* trending in Fig. 4 was mainly due to the increase of the light during the analysis. This can also be observed in micrographs of the flocs at 100× and 200× in Supplementary Material A3 and in Supplementary Material A4. Also, the fact that chlorophyll *a* was increasing while biomass decreased is an interesting pattern observed in this study, especially during the first days. As Adams et al. (2021) referred, chlorophyll *a*-to-biomass ratio varies within and among environments, with several biotic and abiotic factors; even latitude, nutrients, light, and temperatures can influence this parameter. For instance, photosynthetic biomass could have increased over other bacteria and grazers in the inoculum during the first days of operation. Another hypothesis for this phenomenon is the adaptation of the culture to the conditions provided by CWW, which led to floc formations (to be discussed in “Floc formation” section). Overall, the biomass maintained high values despite the low DW of 25%, which is considered a balanced source of nutrients and carbon for microalgae growth (Arias et al. 2021). The results obtained in this work are similar to those reported by Nayak et al. (2016), where 0.196 g L^−1^ day^−1^ of biomass was obtained from *Scenedesmus* sp. cultivated in 100% of DW supplemented with 2.5% (v/v) of CO_2_. It is worthy of note that although the high alkalinity of the CWW provides a carbon source for the culture, the high pH reached along the experimentation indicated carbon deficiency, with photosynthetic activity more intense than respiration. In such a situation, CO_2_ addition could avoid nutrient stripping and increase microalgal growth. In the case of toxicity of industrial effluents, Ajitha et al. (2019) evaluated the toxic effects of an industrial electroplating effluent on *Chlorella vulgaris* and reported a decrease in the content of the photosynthetic pigments (chlorophyll *a*, chlorophyll *b,* and carotenoids) as the effluent concentration in the medium increased. In another remarkable study, Taştan et al. (2017) cultivated cyanobacteria *Geitlerinema* sp. and *Chlorella* sp. in a mix of 25% of boiler cooling water, 25% of Sakarya River water, and 50% of BG11 with different concentrations of toxic triclosan pesticide to evaluate the biodegradation of this contaminant. While *Geitlerinema* sp. showed the highest biomass concentration at low triclosan addition, the microorganisms showed lower tolerance to high concentrations of 11 mg/L of triclosan compared to *Chlorella* sp..

### Carbohydrate production and C/N ratios

**Fig. 5:**
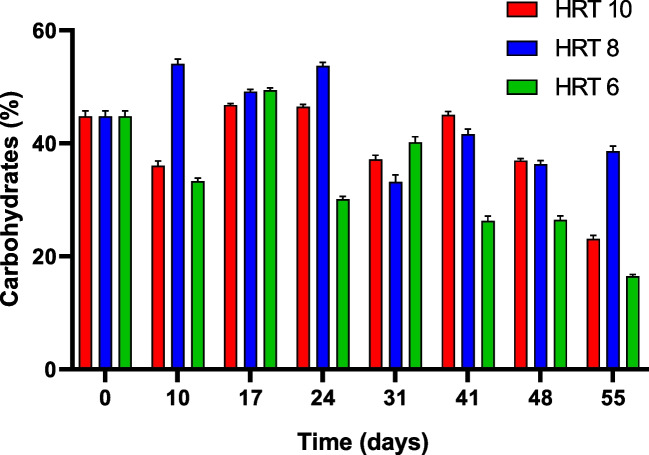
Carbohydrate content in the photobioreactors operated at 10, 8, and 6 days of HRT fed with 25% of domestic wastewater and 75% of cooling wastewater (DW25%-CWW75%)

As the inoculum came from carbon-enriched BG-11 medium, the inoculum presented 45% DCW of carbohydrates at the moment to start the experiment (day 0) (Fig. 5). Subsequently, the three photoreactors showed an increase trending between days 10 and 24 of operation, achieving the highest carbohydrate content of 47%, 54%, and 49% at 10, 8, and 6 days of HRT. After day 24, the percentage of carbohydrates remained below 40% in all reactors: from 23 to 45% DCW in HRT 10, 38% in HRT 8, and 16% on the last day of operation in HRT 6. Also, ANOVA and subsequent *F* test showed statistical differences between all the photobioreactors (*P* = 0.036).

The C/N mol/mol ratios from N nutrients (TAN, nitrates, and nitrites) and inorganic carbon (alkalinity) are presented in Table 3. The results showed unbalanced C/N ratios, showing values ranging from 16.98 to 187.55 in the three photobioreactors, which is very high considering that the optimal C/N ratio for carbohydrate accumulation in microalgae is 6.625 mol/mol (Redfield 1958; Arias et al. 2021). The highest carbohydrate content was obtained when the C/N ratio was below 49.8 mol/mol. Another critical parameter is the N/P ratio. In this study, the highest carbohydrate accumulation was registered at N/P ratios above the standard ratio of 16:1. After day 26, all the N/P ratios were below the standard ratio, which could have induced a low carbohydrate content.

**Table 3:** Ratios C/N and N/P (mol/mol) in the effluent (mixed liquor) of the photobioreactors operated at 10, 8, and 6 days of HRT fed with 25% of domestic wastewater and 75% of cooling wastewater (DW25%-CWW75%)

	HRT 10		HRT 8		HRT 6	
Day	C/N	N/P	C/N	N/P	C/N	N/P
10	16.98	23.94	31.24	12.81	20.53	11.66
17	22.66	28.25	33.80	9.23	44.78	11.88
26	49.80	21.38	46.28	11.64	43.16	6.04
47	144.46	1.25	187.55	0.80	103.63	1.57
55	151.13	1.22	118.69	2.19	30.42	6.39

Although several factors may affect microalgal growth, photosynthetic activity, and carbohydrate accumulation, including trophic modes, nutrient starvation or repletion, pH, temperature, salinity, and light conditions, N limitation with inorganic carbon availability is usually the most common strategy (Debnath et al. 2021). Unbalanced C/N through the experiment showed an evident N deficiency, but affecting the biomass, Chl a, and carbohydrates content differently. During the first days of operation, biomass (measured as VSS) declined as carbohydrate content increased (Figs. 4 and 5). Pearson analysis revealed a moderate correlation between both parameters in the photobioreactor operated at 10 days of HRT and a low correlation at 8 days and 6 days of HRT. After day 26, this correlation was higher in the three conditions when comparing carbohydrates, chlorophyll *a*, and VSS. Considering this result, N/P ratios below 16:1 and the carbon deficiency evidenced by high pH in the second half of the experimental time, maintenance, and increase of VSS and chlorophyll *a* during this period could likely be attributed to carbohydrate consumption by microalgae to meet carbon needs.

Overall, the highest carbohydrate content during the first half of the experimental time, especially in HRT 8, is similar to other carbohydrate contents reported in semi-continuous studies employing domestic wastewater (Table 4). As observed, various authors suggest adding external sources of carbon, such as CO_2_; however, this increases the complexity of the process and operational costs, so using a source of residual nutrients achieves a more sustainable approach (Rueda et al. 2020). In this study, the carbohydrate content is only slightly lower than the content reported in studies that added external carbon sources. However, future studies can be directed towards adding CO_2_ sources to this to know whether carbohydrate content could be improved.

**Table 4:** Comparison of C/N ratio, N/P ratio, and carbohydrate content with other studies

Operating mode	Wastewater	Carbon source	Operation days	HRT (day)	Ratios C/N	Ratios N/P	Maximum carbohydrate content (%)	References
Semicontinuous	CWW-DW	N/A	55	10	22.66	28.25	46.8	**This study**
				8	31.24	12.81	54.1	
				6	44.78	11.88	49.4	
Semicontinuous	Industrial	N/A	35	10	2.5	9	54	(Sánchez-Contreras et al. 2021)
				8	1.65	22	57	
				6	0.76	204	40	
Semicontinuous	DW	N/A	30	10	0.44	37	57	(Solís-Salinas et al. 2021)
				6	0.23	65	56	
Semicontinuous	DW	N/A	30	10	3.67	2.04	48	(Arias et al. 2020)
Semicontinuous	DW	N/A	120	10	NA	0.4	22	(Arcila and Buitrón 2016)
				6	NA	1.1	16	
				2	NA	18.7	14	
Semicontinuous	Syntethic medium (BG-11)	CO_2_ at 10%	16	4	-	-	78	(Yuan et al. 2018)
Continuous	Aquaculture	CO_2_	10	1	-	34.9	-	(Gao et al. 2016)
Semicontinuous	Agricultural runoff	CO_2_ and NaHCO_3_	7 months	15	31.37	46.31	69	(Rueda et al. 2020)
Batch	Syntethic medium (Basal A)	CO_2_ and NaHCO_3_	11	-	3.42	45.7	60	(De Farias Silva et al. 2017)

### Micronutrient content in microalgal biomass

Table 5 shows the composition of nutrients in the harvested algal biomass compared to the stock photobioreactor. The composition of macronutrients such as oxygen and carbon was similar in all PBRs (30–40%), while nitrogen and phosphorus were present in less than 2% in HRT 10, 8, and 6. The marked limitation of these nutrients can explain this behavior during operation, while the stock reactor presented slightly higher N and P amounts (1–7%). The most important micronutrients present in all the PBRs were Ca and Si, ranging from 11 to 26% and 2 to 4%, respectively. These elements were not observed in the stock reactor, implying that the wastewater was the source of these minerals. Particularly, the CWW from the cement industry contains oxides of Ca, Si, Fe, and Al (Venegas Padilla et al. 2017).

**Table 5:** Micronutrient composition in microalgal biomass

Nutrients	Composition (%)			
Elements	Stock reactor	HRT 10 days	HRT 8 days	HRT 6 days
C	43.92 (6.89)	40.63 (13.03)	36.34 (9.92)	35.79 (4.74)
O	36.96 (4.55)	36.93 (5.39)	29.50 (9.37)	37.60 (2.74)
N	6.38 (1.53)	0.00 (0)	1.51 (2.61)	0.00 (0)
P	1.11 (1.25)	0.20 (0.23)	0.79 (0.87)	0.40 (0.39)
S	0.40 (0.49)	0.59 (0.41)	1.16 (0.5)	0.32 (0.26)
K	0.91 (0.84)	1.32 (0.54)	1.08 (0.47)	0.95 (0.37)
Ca	1.53 (2.14)	11.16 (8.54)	26.49 (19)	16.94 (11.3)
Si	0.25 (0.57)	2.77 (2.38)	1.82 (0.61)	3.48 (2.08)
Na	7.83 (3.18)	1.97 (1.21)	0.93 (0.5)	0.51 (0.46)
Cl	0.03 (0.07)	1.26 (0.9)	0.00 (0)	0.00 (0)
Fe	0.00 (0)	1.42 (1.64)	0.00 (0)	1.82 (1.37)
Mg	0.48 (0.46)	1.36 (1.8)	0.38 (0.33)	0.95 (0.59)
Al	0.20 (0.46)	0.40 (0.79)	0.00 (0)	1.23 (0.87)

The metallic micronutrients reported in this analysis (K, Mg, Ca, Na, and Fe) are essential for microalgae since they help regulate cellular functions such as protein and chlorophyll synthesis, osmotic regulation, and nitrogen assimilation (Beltrán-Rocha et al. 2017). Moreover, N, P, S, and K in the residual biomass could be used for agricultural purposes. A similar study by Wuang et al. (2016)) showed that *Spirulina platensis* cultivated in wastewater contained 7.8% of N, 0.8% of P, 1.6% of K, and 0.4% of Ca. Due to the presence of such nutrients, the algal biomass was successfully utilized as a fertilizer for different vegetables.

### Floc formation

Harvesting is one of the greatest challenges in the cultivation of microalgae. It is estimated that around 20 to 30% of the total production costs are allocated only in this stage. Harvesting methods include chemical, physical, or biological flocculation, which is usually carried out by either sedimentation, filtration, centrifugation, or flocculation (Markou et al. 2012; Iasimone et al. 2021). Among these methods, algal biofloculation, which refers to the natural flocculation induced by the agglomeration and sedimentation of various microorganisms such as algae, bacteria, fungi, and yeasts, or bioflocculants, such as extracellular polymeric substances (EPSs) produced by these microorganisms, is considered as the most economically promising (Li et al. 2020a). Bioflocculation is influenced by the physicochemical properties of the mixture, such as the properties of the cell surface, cell concentration, pH, and ionic strength (Krishna 2021).

**Fig. 6:**
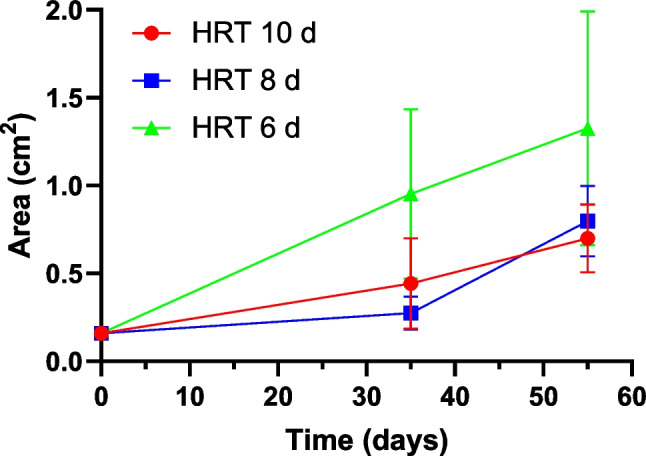
Floc size in cm^2^ along the time in the photobioreactors operated at 10, 8, and 6 of HRT when fed with 25% of domestic wastewater (DW) and 75% of cooling wastewater (CWW)

In this work, bioflocculation occurred naturally over time, without adding any flocculants, resulting in fast settling once agitation was stopped. As shown in Fig. 6, floc size increased with time from the initial average value of 0.16 cm^2^. After 55 days of cultivation, the largest floc size was found in the photoreactor at 6 days of HRT, averaging a value of 1.3 cm^2^ (floc sizes at HRT of 10 days and 8 days were 0.7 and 0.8 cm^2^, respectively). Statistical analysis demonstrated a significant difference between the floc size of the photobioreactors operated at 6 days and 8 days of HRT (*P* = 0.005), but did not show a statistical difference between 6 days and 10 days of HRT (*P* = 0.08). Floc coloration also changed from yellow-green on day 35 to blue-green on day 55 (Supplementary material A4).

Markou et al. (2012) suggested that good settling properties were associated with high carbohydrate content. However, floc formation in this study was not proportional to carbohydrate increase (Figs. 5 and 6). Hence, we hypothesize that other factors such as the HRT and biomass micronutrient content could have promoted the settling properties. Other studies have related good settling properties to bubbling intensity (Iasimone et al. 2020), high bacteria content (Jiang et al. 2021), and hydraulic retention time. For instance, Arcila and Buitrón (2016) studied the effect of different HRT on the flocculation properties of a mixed culture composed of diatoms, green filamentous microalgae, and bacteria cultivated in municipal wastewater. The authors reported that HRT influenced the morphology and structures of biomass cells, associating high HRT with good settling properties. According to them, high HRT promotes a granular morphology that favors floc size and settleability, which is in agreement with our findings. Micronutrient content in the biomass could also affect the flocculation process (Salim et al. 2011). For example, Papazi et al. (2010) evaluated the effect of different salts in the coagulation properties of microalgal cultures of *Chlorella minutissima*, finding that Al^3+^ and Fe^3+^ favored the coagulation process. The natural bioflocculation characteristics observed in this study highlight the benefits of CWW as a cultivation medium for microalgae not requiring the use of coagulants for harvesting.

## Conclusions

The potential of CWW from the cement industry mixed with domestic wastewater (DW) to provide the nutrients to grow a cyanobacteria-dominated culture in medium-term, semi-continuous operation was demonstrated. The results showed high COD and macronutrient removals, while heavy metals were below the limits established by local standards. This study showed that C/N and N/P ratios conditioned biomass growth and carbohydrate content. High alkalinity and micronutrients such as Ca and Si could influence the formation of big flocs, enhancing natural biomass settling and facilitating harvesting. This process represents an alternative for CWW treatment and valorization and a sustainable tool for generating easy-settling carbohydrate-rich biomass with the potential to produce biofuels and fertilizers.

### Supplementary information

ESM 1 (DOCX 2729 kb)

## Data Availability

Not applicable.
