# Supplementary material for: Cultivation of carbohydrate-rich microalgae with great settling properties using cooling tower wastewater
Source: Environ Sci Pollut Res Int. 2023 Jul 6;31(27):38999–9014. doi: 10.1007/s11356-023-28432-w (PMC11186883; doi:10.1007/s11356-023-28432-w)
Supplement: Supplementary file 1 — (DOCX 2729 kb) [file 11356_2023_28432_MOESM1_ESM.docx]

Supplementary material A1. Carbohydrate content in terms of dry cell weight in a batch experiment submitted to different dilutions of domestic wastewater (DW) and cooling water waste (CWW) (v/v) (Ortíz-Sánchez *et al.*, 2022).

Supplementary material A2. Average (standard deviation) of heavy metals in the effluent (mixed liquor) of the photobioreactors.

|  | TRH 10  (mg L^-1^) | Removal (%) | TRH 8  (mg L^-1^) | Removal (%) | TRH 6  (mg L^-1^) | Removal (%) |
| --- | --- | --- | --- | --- | --- | --- |
| **Al** | 0.05 (0) | 54.5 | 0.06 (0) | 45.4 | 0.04 (0) | 63.6 |
| **Cd** | 0.08 (0) | - | 0.26 (0.16) | - | 0.12 (0) | - |
| **Cr** | 0.00 (0) | 100 | 0.00 (0) | 100 | 0.00 (0) | 100 |
| **Cu** | 0.01 (0) | 50 | 0.11 (0) | - | 0.01 (0) | 50 |
| **Fe** | 1.03 (2.79) | 57.9 | 1.07 (2.53) | 56.3 | 1.06 (3.3) | 56.7 |
| **Mn** | 0.00 (0) | 100 | 0.00 (0) | 100 | 0.00 (0) | 100 |
| **Ni** | 0.05 (0) | 16.7 | 0.19 (0) | - | 0.05 (0) | 16.7 |
| **Pb** | 0.43 (0) | 30.6 | 0.41 (0) | 33.8 | 0.34 (0) | 45.1 |
| **Zn** | 0 (0) | - | 0.00 (0) | - | 0.00 (0) | - |


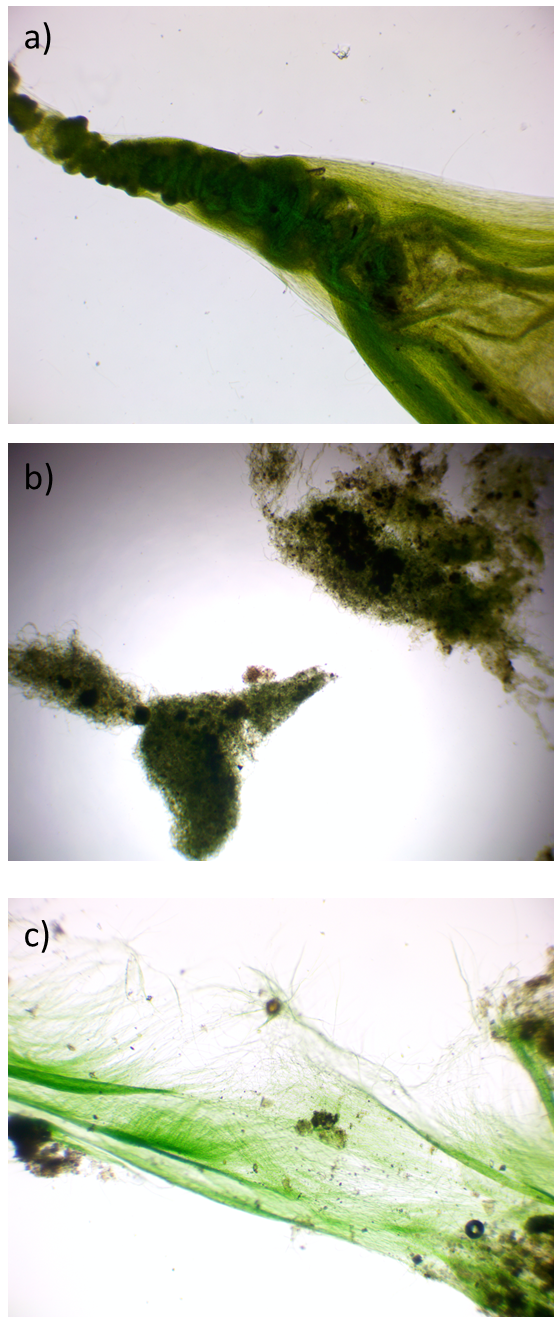


Supplementary material A3. Micrographs of the flocs observed in bright light microscopy at 100× (a, b) and 200x (c) in photobioreactors operated at a) 10, b) 8, and 6 days of HRT fed with 25% of domestic wastewater and 75% of cooling wastewater (DW25%-CWW75%),

***
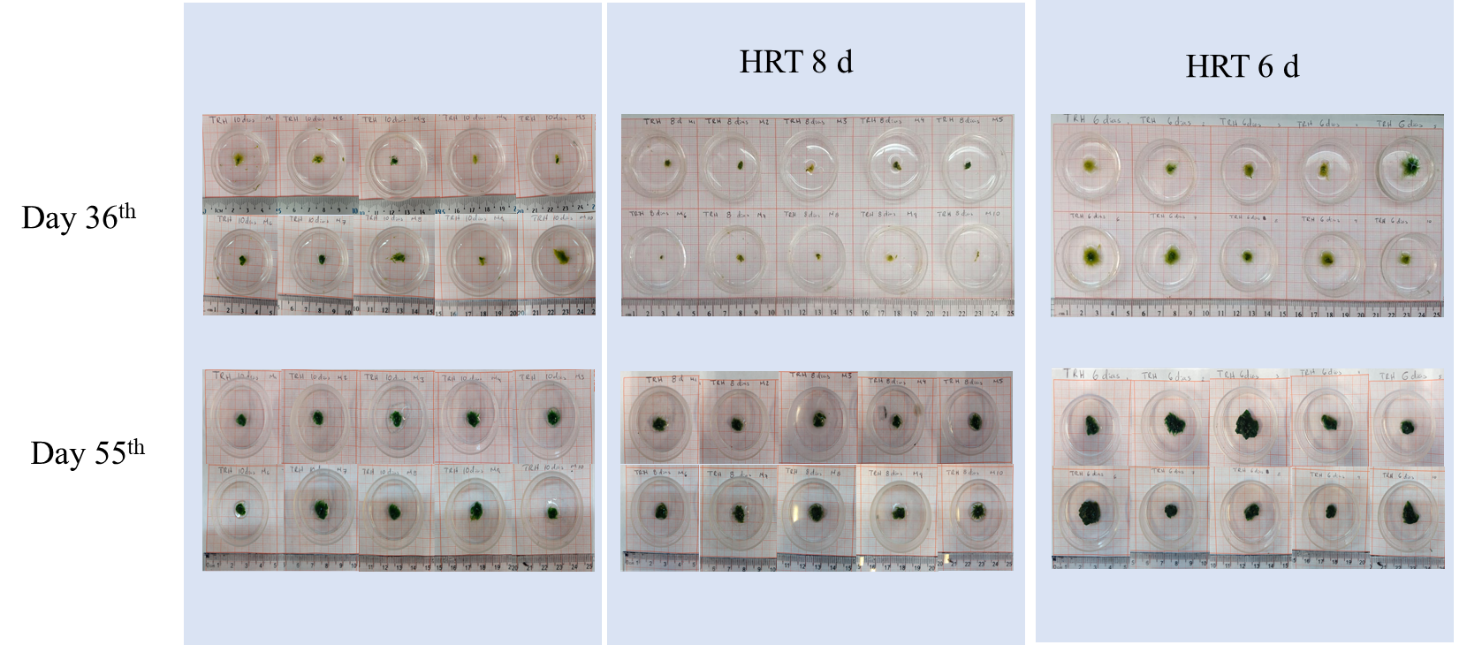
***

Supplementary material A4. Images illustrating the floc size at 10, 8 and 6 of hydraulic retention time in day 36 and 55 of operation.
